# Development of a Humanized HLA-A2.1/DP4 Transgenic Mouse Model and the Use of This Model to Map HLA-DP4-Restricted Epitopes of HBV Envelope Protein

**DOI:** 10.1371/journal.pone.0032247

**Published:** 2012-03-05

**Authors:** Zhitao Ru, Wenjun Xiao, Anthony Pajot, Zhihua Kou, Shihui Sun, Bernard Maillere, Guangyu Zhao, David M. Ojcius, Yu-chun Lone, Yusen Zhou

**Affiliations:** 1 State Key Laboratory of Pathogens and Biosecurity, Beijing Institute of Microbiology and Epidemiology, Beijing, China; 2 INSERM U1014 (ex U542), Université Paris-Sud, Hôpital Paul Brousse, Villejuif, France; 3 Commissariat à l'Energie Atomique-Saclay, Institut de Biologie et Technologies, Service d'Ingénierie Moléculaire des Protéines, Gif-sur-Yvette, France; 4 Health Sciences Research Institute and School of Natural Sciences, University of California Merced, Merced, California, United States of America; King's College London, United Kingdom

## Abstract

A new homozygous humanized transgenic mouse strain, HLA-A2.1^+/+^HLA-DP4^+/+^ hCD4^+/+^mCD4^−/−^IAβ^−/−^β2m^−/−^ (HLA-A2/DP4), was obtained by crossing the previously characterized HLA-A2^+/+^β2m^−/−^ (A2) mouse and our previously created HLA-DP4^+/+^ hCD4^+/+^mCD4^−/−^IAβ^−/−^ (DP4) mouse. We confirmed that the transgenes (HLA-A2, HLA-DP4, hCD4) inherited from the parental A2 and DP4 mice are functional in the HLA-A2/DP4 mice. After immunizing HLA-A2/DP4 mice with a hepatitis B DNA vaccine, hepatitis B virus-specific antibodies, HLA-A2-restricted and HLA-DP4-restricted responses were observed to be similar to those in naturally infected humans. Therefore, the present study demonstrated that HLA-A2/DP4 transgenic mice can faithfully mimic human cellular responses. Furthermore, we reported four new HLA-DP4-restricted epitopes derived from HBsAg that were identified in both vaccinated HLA-A2/DP4 mice and HLA-DP4-positive human individuals. The HLA-A2/DP4 mouse model is a promising preclinical animal model carrying alleles present to more than a quarter of the human population. This model should facilitate the identification of novel HLA-A2- and HLA-DP4-restricted epitopes and vaccine development as well as the characterization of HLA-DP4-restricted responses against infection in humans.

## Introduction

Protective immunity requires the effective mobilization of B cells, cytotoxic T cells and helper T cells [1]. Major Histocompatibility Complex (MHC) molecules play a pivotal role in shaping both the specificity and the functional outcome of adaptive immune responses. The repertoire of functional T cell receptors on CD4^+^ and CD8^+^ T cells is dependent upon the presence of MHC molecules implicated in the positive and negative selection of thymocytes in the thymus [2]. Moreover, the MHC controls the activity of T cells through peripheral tolerance mechanisms and the ability to select and present MHC-restricted epitopes [3].

HLA-restricted epitopes was shown to be important for boosting the effectiveness of HLA-restricted epitope-based vaccine candidates. There is considerable interest in inducing specific CTLs to prevent or control viral infections or to cure autoimmune diseases. Promising protective antiviral immunity using CTL epitope-based vaccines has been demonstrated in several experimental models of infection [4]. Similarly, CD4^+^ T cell epitope-based vaccines have been designed for several experimental autoimmune and pathogenic diseases [5]–[8]. However, these vaccines did not induce significant or sufficient protection in human preclinical trials. The insufficient efficacy could be due to the lack of simultaneous activation of specific B cells, cytotoxic T cells and helper T cells, which is required for protective immunity [9]. Given the practical difficulty of studying immune responses in humans, several transgenic mouse models expressing human HLA class I or class II molecules have been developed to map potential pathogenic and tumoral epitopes as well as to predict human immunity [10], [11].

To facilitate the development of a new generation of candidate vaccines and to evaluate their efficacy *in vivo*, a predictive preclinical “humanized” transgenic mouse model that expresses both HLA-A2 and HLA-DR1 while lacking H2-IAβ and H2-β2m was developed [12]. These mice allow for the simultaneous evaluation of specific B cells, HLA class I-restricted CD8^+^ T cells and HLA class II-restricted CD4^+^ T cells of human interest. However, this humanized mouse model represents the immunity of approximately 3–9% of the human population (30–50% for HLA-A2.1, 6–18% for HLA-DR1) [13], thus limiting the usefulness of this model for preclinical research.

The HLA-DP4 locus is one of the most abundant HLA alleles worldwide (20–80% of the population), being even more abundant than HLA-A2 (30–50%). It consists of two subtypes, DP0401 and DP0402, which differ from each other by 3 amino acids. Preliminary reports have shown that the presence of both DP0401 and DP0402 occurs at a frequency of 50% in Europe, 60% in South America, 80% in North America, 60% in India, 40% in the Xinjiang district in China, 25% in Africa and 15% in Japan [14], [15]. Interestingly, some reports have suggested that antigen presentation by HLA-DP4 molecules may be critical for viral elimination and that such antigen presentation plays an important role in the pathogenesis of chronic hepatitis B infection [16], [17]. These studies highlight the importance of HLA-DP4 in the immune response to infection and in some autoimmunity diseases [18], [19]. Increasing evidence of the importance of HLA-DP4 and its high frequency in the human population suggests that HLA-DP4 epitope-based vaccines could induce immune protection in a large proportion of the population. However, a limited number of HLA-DP4-restricted epitopes have been documented for pathogenic infection [20]–[25] and tumors [26]–[28].

In this study, we describe a homozygous humanized HLA-transgenic mouse strain, HLA-A2.1^+/+^HLA-DP4^+/+^hCD4^+/+^mCD4^−/−^IAβ^−/−^β2m^−/−^ (HLA-A2/DP4), which was produced by crossing the previously characterized HLA-A2^+/+^β2m^−/−^ (A2) mouse [29] and an HLA-DP4^+/+^hCD4^+/+^mCD4^−/−^IAβ^−/−^ (DP4) mouse strain [30] that we established in our laboratory. We then confirmed the phenotypes and immunological activities characteristic of the transgenes (HLA-A2 and HLA-DP4) inherited from the parental HLA-A2 and HLA-DP4 mice. Finally, we screened for HLA-DP4-restricted epitopes derived from HBV envelop protein using the HLA-A2/DP4 mouse, and we identified 4 new HLA-DP4-restricted epitopes (S_256–268_, S_326–338_, S_347–358_ and S_352–364_) that could trigger T helper cell responses similar to those observed in vaccinated HLA-A2/DP4 mice and HBV-vaccinated DP4-positive human donors. Our results demonstrate that the HLA-A2/DP4 mouse represent a promising preclinical research animal model that shares immunological traits with approximately one-quarter of the human population (30–50% for HLA-A2 and 20–80% for HLA-DP4). Thus, this animal model should facilitate the identification of novel HLA-A2- and/or HLA-DP4-restricted epitopes that could be further developed as biomarkers and vaccine components.

## Materials and Methods

### Generation of transgenic mice

HLA-DP4/hCD4 transgenic H-2 class II (IAβ)/mCD4-KO mice [30] were obtained at the INSERM by crossing our established HLA-DP4-transgenic mice (HLA-DP4^+/+^ hCD4^+/+^mCD4^−/−^) with H-2 class II (IAβ)-KO (IAβ^−/−^) mice. The HLA-A2.1-transgenic mice, expressing a chimeric monochain (HHD molecule: α1–α2 domains of HLA-A2.1, α3 to the cytoplasmic domains of H-2 D^b^, linked at its N-terminus to the C-terminus of human β2m by a 15-amino-acid peptide linker), were created in Pasteur Institute [29]. HLA-A2.1 (HHD)-transgenic H-2 class I (β2m)-KO and HLA-DP4/hCD4-transgenic H-2 class II (IAβ)/mCD4-KO mice were intercrossed. After six generation, the heterozygote progeny with HLA-A2.1-HLA-DP4- hCD4 were selected to continue intercrossing till mCD4^−/−^IAβ^−/−^β2m^−/−^ mice were obtained. Finally, HLA-A2.1^+/+^HLA-DP4^+/+^ hCD4^+/+^ mice with the background of mCD4^−/−^IAβ^−/−^β2m^−/−^ mice were obtained and used for the experiments described in this report. Mice were bred in the animal facilities at INSERM U1014 (Paris, France) under barrier conditions and provided with commercial mouse chow and water ad libitum. All experiments involving mice were performed according to approved protocols and the guidelines of the animal facility of Hospital Paul Brousse (Villejuif, France) under agreement number 94-076-32 and permit number 94–241 from the Directorate of Veterinary Services.

### Genotype identification

The HLA-A2.1 (HHD)-transgenic H-2 class I-KO and DP4-transgenic H-2 class transgenic II-KO (IAβb) mice were identified by PCR. Mice genomic DNA was extracted using genomic DNA isolation protocols. Briefly, tails were digested by incubation with 100 mM NaCl, 50 mM Tris-HCl, pH 7.2, 100 mM EDTA, 1% SDS and 0.5 mg/mL proteinase K (Merck, Darmstadt, Germany) overnight at 56°C, followed by the addition of 250 µL of saturated NaCl solution and isopropanol precipitation. Pellets were washed 2 times with 70% ethanol and resuspended in 100 µL deionized water. After homogenization of the DNA concentration, PCR was used to amplify transgenes with different pairs of forward and reverse primers, as follows:

HHD: 5′CATTGAGACAGAGCGCTTGGCACAGAAGCAG3′, 5′GGATGACGTGA GTAAACCTGAATCTTTGGAGTACGC; DP04α: 5′TAATACAAAGTCTGCAGC TGGC3′, 5′AGCAATGTTAGCCAGCC3′; DP04β: 5′GGGATTGGAAAGAGGCT C3′, 5′GCACTGCCCGCTTCTCC3′; hCD4: 5′TCAGTGCAATGTAGGAGTCCAA G 3′, 5′CACGATGTCTATTTTGAACTCCAC3′; mCD4: 5′GGAGTTGTGGGTGTT CAAAGTG3′, 5′AGAGTTGCTATCCAAGGTCAGGG3′; 5′GCTTCCTCGTGCTTT ACGGTATC3′; β2m: 5′CTGAGCTCTGTTTTCGTCTG3′, 5′CTTAACTCTGCA GGCGTATG3′; 5′CCTGCCGAGAAAGTATCCA3′; and Iaβ: 5′TTCGTGTACCAGTTCATGGG3′, 5′TAGTTGTGTCTGCACACCGT3′, 5′CCTGCCGAGAAAGTATCCA 3′.

### FACS analysis

Splenic cells were separated by Ficoll gradients (GE Lifesciences, Uppsala, Sweden). Cytofluorometry studies were performed on splenocytes using PE-conjugated W6/32 (anti-HLA-ABC; eBioscience, San Diego, CA). Analysis of MHC class II molecule expression was conducted after saturation of Fc receptors with the 2.4G2 mAb using FITC-labeled anti-HLA-DP (B7/21) and PE-labeled anti-CD19. CD4^+^/CD8^+^ positive lymphocytes that were first labeled with APC anti-CD3 (BD Biosciences, San Diego, CA, USA). Portions of the single mCD8^+^, mCD4^+^ and hCD4^+^ lymphocytes were labeled using PE-labeled anti-mouse CD8, FITC-labeled anti-mouse CD4, and PECy7-labeled anti-human CD4. Wild-type C57BL/B6 mice were chosen as a control.

### DNA immunization

The hepatitis DNA vaccine pCMV-S2.S [31] was used to verify the consistency in the *in vivo* cellular responses between the transgenic mice and humans. At the age of 8 weeks, transgenic mice were pre-immunized with cardiotoxin. After 5 days, the mice were immunized 3 times intramuscularly at 10-day intervals, each time with a 100-µg DNA vaccine injection. Ten days after the last immunization, the mice were used for further analyses.

### ELISA assay

Sera from immunized mice were individually assayed by ELISA [31] on either purified HBsAg or preS2 synthetic HBs109–134 peptide. After blocking with PBS supplemented with 0.1% Tween-20, 10% FCS and washings three times, bound antibodies were detected with horseradish peroxidase-labeled anti-mouse IgG (Serotec, Cergy-Saint-Christophe, France). Antibody titers (means of at least three determinations) were determined by the serial end-point dilution method.

### ELISPOT assay

An ELISPOT assay was implemented to detect IFN-γ secreted by CD8^+^ T lymphocytes. Briefly, membrane-backed 96-well ELISPOT plates (Millipore, Bedford, MA) were coated with anti-IFN-γ mAb (Diaclone, Besancon, France) overnight at 4°C and then blocked with 1% skim milk. CD4-lymphocyte-depleted cells (2×10^5^/well) were added to each well, cultured with 20 µg/mL synthetic peptides and incubated for 20 h at 37°C under 5% CO_2_. The IFN-γ-secreting cells were captured by coating with anti-IFN-γ mAb and detected by incubation with biotinylated anti-mouse IFN-γAb (Diaclone) for 90 min at 37°C, followed by incubation with streptavidin-HRP for 1 h. Finally, the plates were developed using substrate BEC (Diaclone, ready to use), washed, and dried. Spots were counted using an ELISPOT reader (CTL, Germany).

### Proliferation assay

Ten days after the final immunization, splenocytes were RBC-depleted, submitted to a Ficoll gradient, and adjusted to 10×10^6^ cells/mL (5×10^5^cells/well) [12]. Splenocytes were co-cultured with peptide-pulsed (20 µg/mL) in HL1 serum-free medium supplemented with 10 mM HEPES, 1 mM sodium pyruvate, 5×10^−5^ M 2-mercaptoethanol, 100 IU/mL penicillin and 100 µg streptomycin for 72 h at 37°C in 5% CO_2_. One microcurie of [^3^H]thymidine was added to each well 16 hours before the cells were harvested using a TOMTEC collector (Perkin Elmer Applied Biosystems), and the incorporated radioactivity was measured using a micro-β-counter (Perkin Elmer Applied Biosystems). The results are given as the stimulation index (SI) = cpm with specific peptide/cpm with control peptide (Mag-3_243–258_ KKLLTQHFVQENYLEY) [32].

### HLA-DP4 specific binding assay

HLA-DP4 binding assays were performed as described elsewhere [14]. In brief, they were performed in 10 mM phosphate, 150 mM NaCL, 1 mM n-dodecyl β-D-maltoside, 10 mM citrate, and 0.003% thimerosal (PH 5) buffer with 10 mM of bOxy271-287 peptide, an appropriate dilution of HLA-DP4 molecules (∼0.1 µg/mL), and serial mid-dilutions of competitor peptides. After 24 h incubation at 37°C, samples were neutralized and applied to B7/21-coated plates for 2 h. Bound biotinylated peptide was detected by means of streptavidin-alkaline phosphatase conjugate (Amersham, Little Chalfont,UK) and 4-methylumbelliferyl phosphate substrate(Sigma-Aldrich). Emitted fluorescence was measured at 450 nm upon excitation at 365 nm in a victor II spectrofluorometer (PerkinElmer Instruments, Les Ulis, France). Data were expressed as the peptide concentration that prevented binding of 50% of the labeled peptide(IC_50_). IC_50_ values of the Oxy_271–287_ peptides served as reference in each experiment.

### Human T cell proliferation assays

Human peripheral blood mononuclear cells (PBMCs) were incubated with the HBsAg peptides and the positive control peptide at 20 µg/mL in T cell medium containing 7.5% AB serum for 5 days and pulsing with one microcurie of [^3^H]thymidine for 16 hours before harvesting. Wells were harvested using the TOMTEC collector (Perkin Elmer Applied Biosystems), and the incorporated radioactivity was measured using a micro-β counter (Perkin Elmer Applied Biosystems). The results are given as the stimulation index (SI) = (cpm with specific peptide)/(cpm with control peptide).

## Results

### Cell surface expression of MHC molecules in HLA-A2/DP4 mice

**Figure 1:**
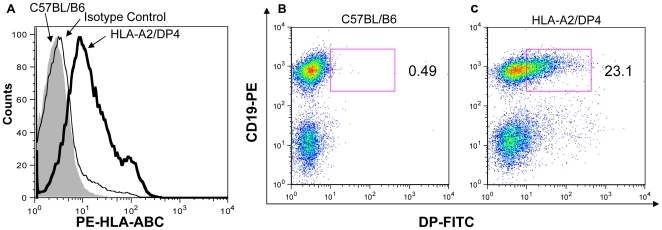
Flow cytometric analysis of HLA-A2 and HLA-DP4 expression of transgenic molecules. (A) Splenocytes from HLA-A2/DP4 (bold histogram) and wild-type C57BL/B6 (shadowed histogram) mice were isolated and stained with PE-labeled anti-HLA-ABC mAb to observe the HLA-A2 expression. PE-labeled mouse IgG2a, k mAb was used as isotype control (thinned histogram) for staining HLA-A2/DP4 mouse. (B) Splenocytes from wild-type C57BL/B6 mice (Figure 1B) and HLA-A2/DP4 (Figure 1C) were isolated and stained with PE-labeled anti-CD19 and FITC-labeled anti-HLA-DP mAb to observe the HLA-DP4 expression.

HLA-A2/DP4 mice were obtained by crossing the parental HLA-A2^+/+^β2m^−/−^ (A2) mice and HLA-DP4^+/+^hCD4^+/+^mCD4^−/−^IAβ^−/−^ (DP4) mice. The genotype and cell surface expression of HLA-A2.1 and HLA-DP4 were confirmed on splenocytes from the HLA-A2/DP4 mice by PCR (data not shown) and flow cytometry. As illustrated in Figure 1A, HLA-A2.1 expression was observed in HLA-A2/DP4 mice, whereas no expression was detected in wild-type C57BL/B6 mice. As shown in Figure 1B and 1C, HLA-DP4 expression was observed only in the HLA-A2/DP4 mice(Figure 1C), however, no HLA-DP4 expression was detected as expected (Figure 1B). In addition, DP4+CD19− T lymphocytes were further analyzed by staining PEcy7-labeled anti-CD11b and FITC-labeled anti-CD11c in Figure S1.

### Peripheral CD8^+^ T and CD4^+^ T lymphocytes in HLA-A2/DP4 mice

**Figure 2:**
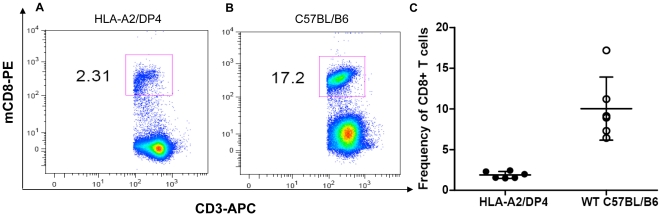
Flow cytometric analysis of peripheral CD8^+^ T lymphocytes. Splenocytes from HLA-A2/DP4 (Figure 2A) and wild-type C57BL/B6 mice (Figure 2B) were isolated and CD3^+^ T cells were gated by staining APC-conjugated anti-CD3 mAb and CD8^+^ T cells were gated by staining PE-conjugated anti-mCD8 mAb. Frequencies of CD8^+^ T cells among total CD3^+^ T cells from 6 mice of each group were shown in Figure 2C.

**Figure 3:**
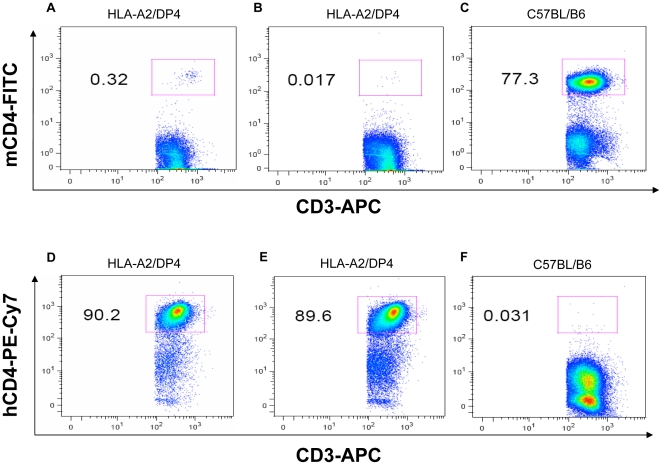
Flow cytometric analysis of peripheral mCD4^+^ and hCD4^+^ T lymphocytes. Splenocytes from HLA-A2/DP4 (Figure 3A, 3B, 3D and 3E) and wild-type C57BL/B6 (Figure 3C and 3F) mice were isolated and CD3^+^ T cells were gated by staining with APC-conjugated anti-CD3 mAb. Meanwhile, FITC-conjugated anti-mCD4 and PECy7-conjugated anti-hCD4 antibodies were simultaneously used to observe the mCD4 and hCD4 expression in HLA-A2/DP4 and WT C57BL/B6 mice.

Splenic CD4^+^ and CD8^+^ T cell numbers were determined by immunostaining and flow cytometry analysis. T lymphocytes were labeled with APC-conjugated anti-CD3^+^ antibody and then with PE-conjugated anti-mCD8^+^, FITC-conjugated anti-mCD4^+^ and PECy7-conjugated anti-hCD4^+^ antibodies, as illustrated in Figures 2–3.

The expression of HLA-A2.1 and HLA-DP4 molecules allowed for the selection of peripheral CD8^+^ and CD4^+^ T cells in HLA-A2/DP4 mice. As shown in Figure 2, around 2.3% CD8^+^ T cells (Figure 2A and 2C), compared with 0.6–1% CD8^+^ T cells in β2-microglobulin (β2m)-deficient MHC class I-deficient mice [29]; there were 89–90% CD4^+^ T cells (Figure 3D and 3E) compared with 2–3% CD4^+^ T cells in the H-2 class II-KO mice [33]. In comparison, wild-type C57BL/B6 mice possess around 17.2% CD8^+^ T cells (Figure 2B and 2C) and 77% CD4^+^ T cells (Figure 3C) in CD3+ T cells.

It has been reported that the differentiation of immature CD4^+^CD8^+^ double-positive T cells into CD4^+^ T cells in the thymus is highly dependent on the expression of human CD4 (hCD4) [34]. As shown in Figures 3D and 3E, 89–90% of CD4^+^ T cell expressed hCD4, and 0–0.3% of the remaining cells exhibited nonspecific staining with FITC-labeled anti-murine CD4 (mCD4) in HLA-A2/DP4 mice (Figure 3A and 3B). In contrast, 77.3% of the CD4^+^ T cells in wild-type C57BL/B6 mice C57BL/B6 mice express mCD4 (Figure 3C) rather than hCD4 (0.03%) (Figure 3F). In addition, flow cytometric analysis was also used to observe the percentage of Treg cells in CD4^+^ T cells in Figure S2.

### Humoral responses and HLA-A2-restricted responses in HLA-A2/DP4 mice

To evaluate the immunological potential of HLA-A2/DP4 mice and their ability to predict human responses, we compared the humoral response and the immunodominant HLA-A2-restricted response in the HLA-A2/DP4 mice with the responses that have been previously reported for naturally infected patients or immunized humans. We immunized the HLA-A2/DP4 mice with an HBV DNA vaccine that encodes two HBV envelope proteins (preS2 and S proteins). These two proteins can self-assemble into particles carrying HBsAg, and they are the two protein components of currently used vaccine against hepatitis B.

**Figure 4:**
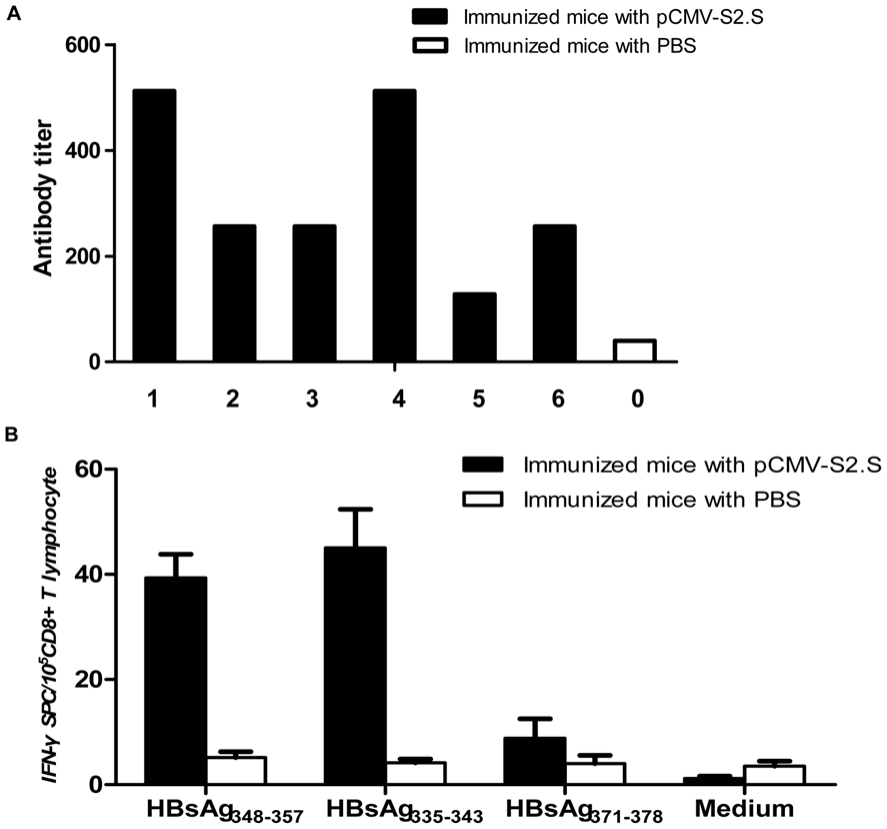
HBs-specific antibody and cytotoxic responses. (A) Sera were collected 10 days after the third immunization, and the titers of the antibody (IgG) against HBs particles in the immunized mice (solid bar) were determined and compared with the mean responses of six PBS-immunized mice (hollow bar) in an ELISA assay. (B) HBs epitope-specific IFN-γ production by CTLs was determined by measuring response to the HLA-A2-restricted epitopes HBsAg348–357 and HBsAg335–343 and to the H-2 K^b^-restricted epitope HBsAg371–378 in immunized mice (solid bar) and PBS-immunized mice (hollow bar).

To show that the CD8^+^ T cells in the periphery of HLA-A2/DP4 mice are functionally restricted by transgenic human class I molecules (as reported for the transgenic HLA-A2 mice [29]), we examined the HBsAg-specific CD8^+^ CTL responses by monitoring the immunodominant HLA-A2.1-restricted epitope responses directed against HBsAg_348–357_ [35] and HBsAg_335–343_ [36] epitopes. The results showed that after immunization with the pCMV-S2.S HBV DNA vaccine, the transgenic mice exhibited HBsAg-specific humoral responses (Figure 4A) and HLA-A2-restricted CD8^+^ T cell responses (Figure 4B). The immunodominant HLA-A2.1-restricted response is directed against the HBsAg_348–357_ and HBsAg_335–343_ epitopes, whereas in wild-type C57BL/B6 mice, the H-2 K^b^-restricted HBsAg-specific CTL response is directed against the HBsAg_371–378_ epitope [37]. To determine whether HLA-A2/DP4 mice mount the same HLA-A2-restricted CTL response as HLA-A2/DR1 mice and humans, splenic T cells were stimulated with the relevant HLA-A2.1-restricted peptides, HBsAg_348–357_, HBsAg_335–343_ and a control (HBsAg_371–378_, H-2 K^b^-restricted), and the secretion of IFN-γ was measured as a read-out. As expected, HBsAg DNA immunization elicited a significant CTL response against HBsAg_348–357_ and HBsAg_335–343_, and there was no response against HBsAg_371–378_ (Figure 4B). The results demonstrate that CD8^+^ T cell responses in HLA-A2/DP4 mice are HLA-A2-restricted and are similar to the CD8^+^ CTL response after immunization of HLA-A2/DR1 transgenic mice with the same vaccine [12].

### HLA-DP4-restricted responses in HLA-A2/DP4 mice

**Figure 5:**
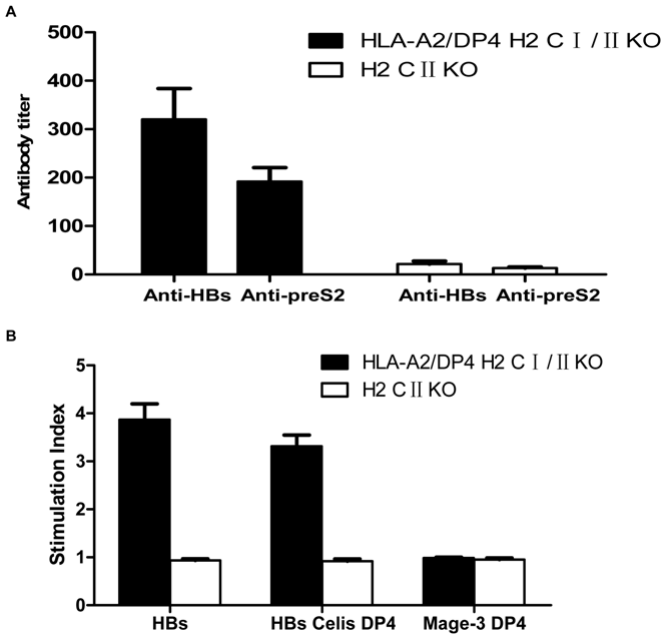
HBs-specific antibody and proliferative response. (A) The titers of antibody (IgG) from HLA-A2/DP4 transgenic mice (solid bar) and H-2 class II-deficient mice (hollow bar) against HBs particles and the preS2_109–134_ peptide were determined using an ELISA assay (Figure 5A). (B) The HLA-DP4-restricted proliferation of CD4^+^ T cells from HLA-A2/DP4 transgenic mice (solid bar) and H-2 class II-deficient mice (hollow bar) after stimulation with HBs particles, the previously described HBV DP4 restricted peptide, S181–S192, or the control HLA-DP4-restricted epitope, Mag-3_243–258_ (Figure 5B).

To evaluate the behavior of CD4^+^ T cells in HLA-A2/DP4 mice, we immunized the mice with a hepatitis B virus DNA vaccine, pCMV-S2.S, by intramuscular injection. H2-class II-deficient mice were used as a control [33]. As shown in Figure 5A, HBs protein- and PreS2 antigen-specific antibodies were induced in HLA-A2/DP4 transgenic mice (solid column) at 10 days after the third immunization. Conversely, no antigen-specific antibodies were detected in H2-class II-deficient mice. This result demonstrates that potent humoral responses require the help of CD4^+^ T cells.

In the *in vitro* proliferation assays (Figure 5B), HBsAg DP4-restricted CD4^+^ T cells responded to whole HBs antigen and to the previously described HLA-DP4-restricted peptide, S_181_–S_192_ [20]. No responses were observed in the H2-class II-deficient mice (hollow column) or to the control HLA-DP4-restricted peptide, Mage-3_243–258_ [32]. The results demonstrate that HBsAg could induce the HLA-DP4-restricted proliferation of CD4^+^ T cells in HLA-A2/DP4 transgenic mice.

### Identification of novel HLA-DP4 epitopes that respond to the HBsAg DNA vaccine

Having documented that the HLA-A2/DP4 mice could respond to HBsAg, a previously described DP4-restricted epitope, we next sought to identify novel HLA-DP4-restricted epitopes in the HBs envelope protein. The immunogenicity of both T helper and CTL epitopes are related to their MHC-binding capacities, with good T helper inducers generally having a high affinity for MHC class II molecules. We therefore scanned the hepatitis B envelope protein based on the HLA-DP4 peptide-binding motif [38] and selected 11 candidate peptides. The affinities for each of the candidate peptides were evaluated (Table 1). We also immunized HLA-A2/DP4 transgenic mice intramuscularly with the pCMV-S2.S DNA vaccine. The splenocytes derived from the primed mice were separated and then stimulated *in vitro* with 12 HLA-DP4-restricted peptides, including the HLA-DP4-restricted epitope HBs_181–192_. The peptides S109–121, S256–268, S326–338, S347–358 and S352–364 induced proliferative responses in HLA-A2/DP4 mice, whereas no responses were observed in the control H-2 class II-deficient mice. Thus, the HLA-A2.1^+/+^HLA-DP4^+/+^hCD4^+/+^ mCD4^−/−^ IAβ^−/−^β2m^−/−^ mice allowed us to identify 5 novel HLA-DP4-restricted epitopes from the hepatitis B envelope protein.

**Table 1:** DP4 epitopes binding ability and proliferative response in HLA-A2/DP4 mice.

	Anchors	Exp	Obs	SI	
Positions	1 6 9	IC50(nM)	IC50(nM)	HLA-A2/DP4	H2 CII KO
S109–121	MQ**W**NSTT**F**HQ**T**LQ	40	144	2	1
S165–177	EN**I**TSGF**L**GP**L**LV	180	>10000	1	1
S181–192	GF**F**LLTR**I**LT**I**PQ	70	2	2–3	1
S197–209	SW**W**TSLN**F**LG**G**TT	300	737	1	1
S256–268	FL**L**VLLD**Y**QG**M**LP	120	>10000	2–3	1
S318–331	SS**W**AFGK**F**LW**E**WA	110	46	1	1
S319–331	WA**F**GKFL**W**EW**A**SA	160	221	1	1
S326–338	WE**W**ASAR**F**SW**L**SL	10	77	2	1
S347–358	VG**L**SPTV**W**LS**V**I	120	30	2–3	1
S352–364	TV**W**LSVI**W**MM**W**YW	28	>1000	2–3	1
S362–374	WY**W**GPSL**Y**SI**L**SP	40	952	1	1
S376–388	LP**L**LPIF**F**CL**W**VY	60	626	1	1

Twelve HLA-DP4-restricted epitopes were predicted by scanning the entire HBs protein and were synthesized. Exp IC50 (nM) represents the predicted affinities according to algorithms, while Obs (nM) represents the affinities obtained from MHC class II binding assays. [^3^H]thymidine incorporation assays were used to measure the proliferation of CD4^+^ T cells from DNA-vaccine-immunized HLA-A2/DP4 and H-2 class II-deficient mice stimulated with the twelve synthesized peptides, including the previously reported epitope, S181–192. Exp = Expected, Obs = Observed, SI, stimulation index.

### HLA-DP4-restricted CD4^+^ T cell responses in vaccinated humans

To determine whether the epitopes that we identified in the HLA-A2/DP4 mice were relevant for human immune responses, PBMCs from 6 HLA-DP4+ donors, including 4 donors who had been immunized against HBV and 2 donors who were never immunized, were utilized to analyze their HLA-DP4-restricted CD4+ T cell responses. The results of the proliferation assays (Table 2) showed that three out of four immunized subjects responded to the reported Celis (S181–S192) peptide. The responses of two donors were directed against the S326–338 and S256–268 peptides. One response was observed against the S352–364 and S347–358 peptides. No significant responses against the S109–121 peptide (which was derived from the S2 part of the antigen) were observed in the four donors who had received the HBV vaccine. All together, four out of five newly identified DP4-restricted HBs epitopes were functional to detect specific T cell responses in vaccinated humans.

**Table 2:** Proliferative response in DP4-positive donors.

	DP4+ HBV vaccinated (SI)				DP4+ unvaccinated (SI)	
Positions	Donor 1	Donor 2	Donor 3	Donor 4	Donor 5	Donor 6
S109–121	1.1	0.9	0.8	1.0	0.8	0.9
S181–192*	7.2	3.6	0.8	3.1	0.8	1.1
S256–268	2.4	1.4	0.4	3.1	0.7	1
S326–338	1.4	2.3	0.5	3.5	1	1
S347–358	1.2	2.2	0.6	1.1	1	0.9
S352–364	1.2	3.1	0.7	1.6	1	0.7

*S181–S192 peptide was used as positive control.

The proliferation of CD4^+^ T cells in 4 HBV vaccinated and 2 unvaccinated HLA-DP4^+^ donors after *in vitro* stimulation with 5 newly identified HLA-DP4-restricted epitopes and one positive control peptide S181–S192. SI, stimulation index.

## Discussion

In the present study, we developed an HLA transgenic mouse model that lacked an H-2 antigen system. As we mentioned above, this newly created HLA-A2/DP4 transgenic mouse model combines the most frequent HLA class I allele, A*0201, with the most frequent class II allele, HLA-DP4. At least a quarter of the Caucasian population (which is ∼50% positive for HLA-A*0201 and ∼76% for HLA-DP4 [13]), for example, carries the HLA-A2/DP4 genotype. Thus, this mouse model could predict human cellular responses for a larger proportion of the human population than our previously established HLA-A2/DR1 mouse model.

According to human allele frequency statistics, DP4 is the most abundant allele in the world, and it shares a motif with other subtypes DPB1*0101, DPB1*0501 and DPB1*0201 [39] as DR and DQ superfamilies. Furthermore, the HLA-DP4 allele has become increasingly valuable in preclinical research. Accumulating evidence has proven that DP4 is important in immunity, and it is a key allele in preventing viral infection, autoimmunity and transplant rejection [40]. A recent study indicated that DP4 is a protective allele in preventing chronic infection with HBV. Other studies have shown that the presence of DP4 is correlated with diabetes and multiple sclerosis [18], [19]. These studies were based on statistical analyses, and further explanation of this mechanism is required. However, polymorphisms among different species and individuals as well as a lack of human samples have hampered HLA studies leading to DP-related research, and there are surprisingly few mouse models for DP.

In the HLA-A2/DP4 mouse model, the development of a repertoire of CTLs and T helper cells and the mobilization of effector cells in the periphery should be restricted to HLA molecules rather than murine H-2 molecules. We confirmed HLA-A2 and DP4 molecules were expressed on the cell surface in HLA-A2/DP4 mouse model. With the regulation of HLA molecules, there is a normal population of T lymphocytes in the periphery. Even through the percentage of CD8 T cells is relatively low in HLA-A2/DP4 mice, this observation in accordance with our previously reported HLA-A2/DR1 mice in 2004. Even in this case, expression of transgenic HLA-A2.1 molecule led to an increase in the size of the peripheral CD8+ T cell population, which reached 2–3% of the total splenocytes in HLA-A2/DR1 mice, compared to 0.6–1% in β2m-KO MHC-I deficient mice [12]. Furthermore, this phenomenon had been discussed and several studies on HBV and HIV proved that the low percentage for CD8+ T cell doesn't significantly affect the usage of HLA-A2/DR1 mice in immunological analysis [41], [42].

Then, we observed the humoral and HLA-restricted immune responses in this mouse model by studying HBV DNA immunization. After DNA immunization, we could detect a significant humoral response against target proteins. Meanwhile, we confirmed that both the CTL response and the Th response were HLA-restricted responses rather than H-2 restricted responses. Our results demonstrated that these mice exhibit HLA-DP4 responses similar to those observed in humans, and the mice should be useful, not only for the identification of new HLA-DP4-restricted epitopes and assessing the efficiency and safety of novel vaccines but also for analysis of the cooperation between HLA-A2 and HLA-DP4 and their contribution to immunity.

Compared with HLA-A2, DP4 restricted epitopes of HBV are rarely reported. Therefore, mapping immunodominant DP4 epitopes will be of great value for vaccine applications. Given that one epitope identified in HLA-A2/DP4 transgenic mice did not induce a significant response in vaccinated humans, it is possible that competition among different HLA-class II molecules in human body results in the selection of immunodominant epitopes that would be concentrated on the human MHC molecules in the humanized mice. In a natural situation, several HLA genes are codominantly expressed in the ER, and they can compete for overlapping epitopes [43]–[45]. Thus, the set of peptides associated with an HLA molecule can be influenced by the presence of other HLA molecules. In addition, we speculate that other factors may have influenced the results, such as Treg cells; it is reported that Tregs epitopes can suppress the proliferation of other T cells after peptide stimulation *in vitro* [46] but this speculation need further verification.

In conclusion, we reported the creation of a new HLA-A2/DP4 mouse model that can be reliably used to identify immunodominant epitopes in humans and to rank their ability to prime CTLs and T helper cells. This mouse model thus represents a promising surrogate model for studying the immune responses before human preclinical trials.

## Supporting Information

Figure S1 **Flow cytometric analysis of HLA-DP4 expression of transgenic molecules.** Splenocytes from wild-type C57BL/B6 mice (Figure S1A) and HLA-A2/DP4 (Figure S1B) were isolated and stained with APC-labeled anti-CD19 and PE-labeled anti-HLA-DP mAb to observe the HLA-DP4 expression. In addition, DP4+ CD19− T lymphocytes were further analyzed by staining PEcy7-labeled anti-CD11b and FITC-labeled anti-CD11c(Figure S1C).
(TIF)

Figure S2 **Flow cytometric analysis of the percentage of Treg cells.** Splenocytes from HLA-A2/DP4 and wild-type C57BL/B6 mice were isolated and CD3+ T cells were gated by staining with FITC-labeled anti-CD3 mAb. Meanwhile, PEcy7-labeled anti-hCD4 mAb and APC-labeled anti-Foxp3 mAb were simultaneously used to observe the Treg frequency in hCD4^+^ T cells of HLA-A2/DP4 mice(Figure S2A, S2B, and S2C), while PECy7-conjugated anti-mCD4 mAb and APC-labeled anti-Foxp3 mAb were simultaneously used to observe the Treg frequency in mCD4^+^ T cells of WT C57BL/B6 mice(Figure S2D, S2E, and S2F).
(TIF)
