# Supplementary figures and images for: Development of a Humanized HLA-A2.1/DP4 Transgenic Mouse Model and the Use of This Model to Map HLA-DP4-Restricted Epitopes of HBV Envelope Protein
Source: PLoS One. 2012 Mar 5;7(3):e32247. doi: 10.1371/journal.pone.0032247 (PMC3293898; doi:10.1371/journal.pone.0032247)

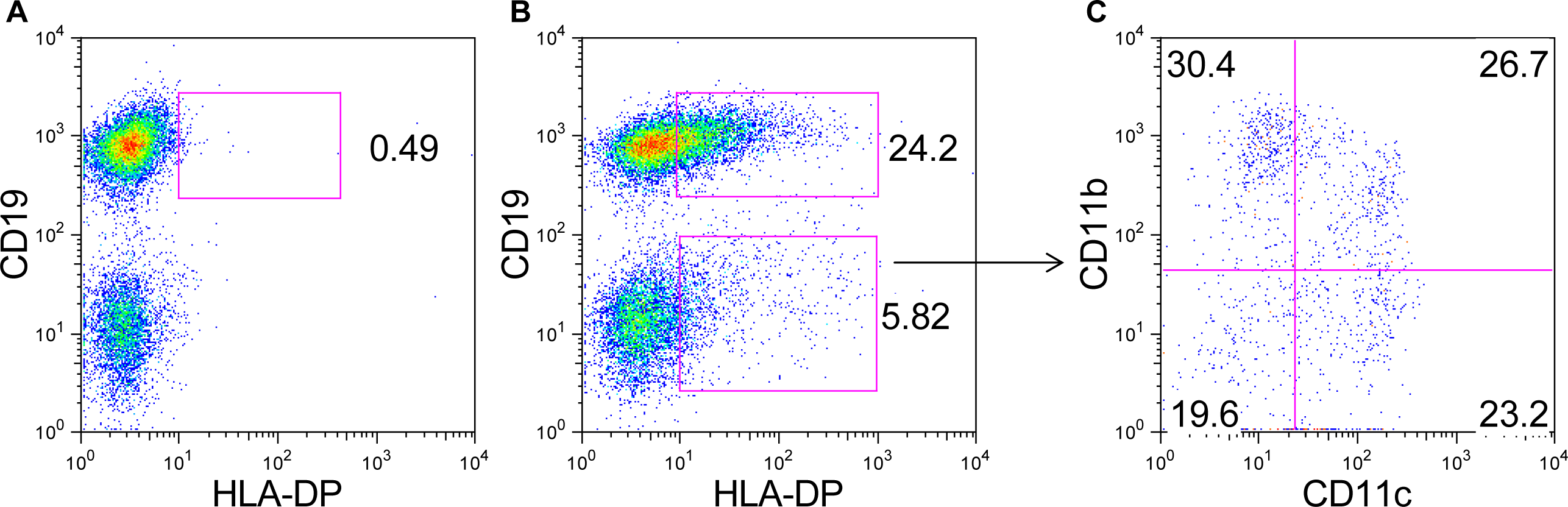

Supplement: Figure S1 — Flow cytometric analysis of HLA-DP4 expression of transgenic molecules. Splenocytes from wild-type C57BL/B6 mice (Figure S1A) and HLA-A2/DP4 (Figure S1B) were isolated and stained with APC-labeled anti-CD19 and PE-labeled anti-HLA-DP mAb to observe the HLA-DP4 expression. In addition, DP4+ CD19− T lymphocytes were further analyzed by staining PEcy7-labeled anti-CD11b and FITC-labeled anti-CD11c(Figure S1C). (TIF) [file pone.0032247.s001.tif]

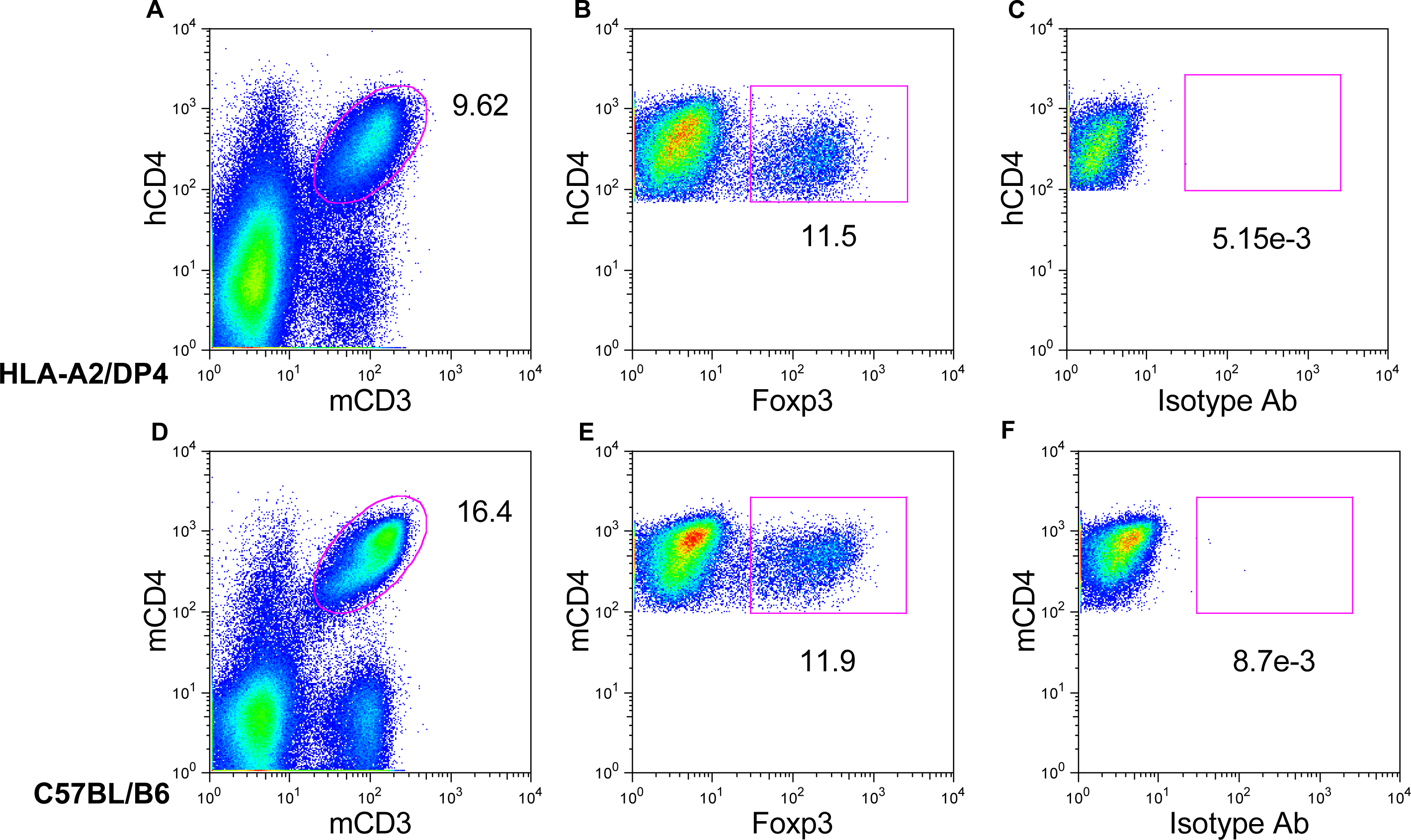

Supplement: Figure S2 — Flow cytometric analysis of the percentage of Treg cells. Splenocytes from HLA-A2/DP4 and wild-type C57BL/B6 mice were isolated and CD3+ T cells were gated by staining with FITC-labeled anti-CD3 mAb. Meanwhile, PEcy7-labeled anti-hCD4 mAb and APC-labeled anti-Foxp3 mAb were simultaneously used to observe the Treg frequency in hCD4+ T cells of HLA-A2/DP4 mice(Figure S2A, S2B, and S2C), while PECy7-conjugated anti-mCD4 mAb and APC-labeled anti-Foxp3 mAb were simultaneously used to observe the Treg frequency in mCD4+ T cells of WT C57BL/B6 mice(Figure S2D, S2E, and S2F). (TIF) [file pone.0032247.s002.tif]
